# Childhood occasional hypertension and its association with size at birth and early growth: a population-based retrospective cohort study from China

**DOI:** 10.1186/s13052-025-01860-9

**Published:** 2025-02-07

**Authors:** Shuang Zhang, Leishen Wang, Tao Zhang, Yijuan Qiao, Wei Li, Weiqin Li

**Affiliations:** Tianjin Women and Children’s Health Center, Tianjin, 300070 China

**Keywords:** Children, Occasional hypertension, Small for gestational age, Body mass index

## Abstract

**Background:**

Hypertension has recently shown a rapid rise in prevalence among children and adolescents. It can track into adulthood and tend to manifest at an earlier age. It should be prevented urgently and efficiently.

**Methods:**

This study assesses the prevalence of occasional hypertension (OHTN) at 5 ~ 6 years old and evaluates its association with size at birth and BMI at 2 and 5 ~ 6 years old in full-term children. We conducted a population-based cohort study of 12,564 children from 66 kindergartens in Tianjin, China. Information on birth weight, body mass index (BMI) at 2 and 5 ~ 6 years old, and blood pressure at 5 ~ 6 years old was retrospectively collected.

**Results:**

The prevalence of childhood OHTN (SBP or DBP ≥ P_95_ percentile for sex, age, and height) was 17.0%. At birth, SGA has a high risk of OHTN (AOR 1.36, 95%CI 1.10–1.68). In the SGA subgroup, although children are of normal weight at 5 ~ 6 years old, excessive catch-up growth (BMI ≥75th at 2 years old) was still attributed to OHTN (OR 1.51, 95%CI 1.03–2.24). Obesity (BMI ≥2SD at 5 ~ 6 years old) was a vital risk factor for OHTN (AOR 2.93, 95%CI 2.56–3.36) unregarding with birthweight (OR 95%CI: SGA 3.23(1.66 ~ 6.27), AGA 2.83 (2.42–3.31), LGA 3.52 (2.65–4.68)). The co-presence of moderate or excessive catch-up growth before 2 years old and obesity at 5 ~ 6 years old significantly increased the risk of childhood OHTN (OR from 2.74 (1.65–4.54) to 6.53 (2.68–15.90)).

**Conclusions:**

Preschool obesity, low birth weight, and excessive catch-up growth increased the risk of OHTN in childhood.

## Background

Hypertension (HTN), also known as elevated blood pressure (BP), is a leading risk factor contributing to cardiovascular disease, morbidity and mortality [1]. In addition to its high prevalence in adults, HTN has recently shown a rapid rise in prevalence among children and adolescents, trending to manifest at an earlier age. The prevalence of HTN was 16.3% in 2017 in the US among adolescents aged 10 ~ 17 years [2] and 9.2% in 2014 in China among children aged 7 ~ 17 years [3]. Childhood HTN can track into adulthood with the phenomenon of ‘trajectory’ and has been suggested by pathophysiologic and epidemiologic evidence to be associated with organ damage, including coronary artery calcifications and hypertrophy [4, 5]. Therefore, the prevention of HTN in children and adolescents should be implemented urgently and efficiently.

BP levels in children are dynamic and can be easily influenced by various factors. Previous studies have indicated that up to half of the children referred for evaluation of elevated office blood pressure have white-coat hypertension (WCH) [6, 7]. In adults, some researchers have documented an imbalance between the sympathetic and parasympathetic nervous systems, showing excessive sympathetic activity [8, 9]. There is evidence suggesting that target organ effects, including those on the heart, kidneys, brain, and vasculature, are present in WCH compared to normotension, but these effects are not as pronounced as those found in hypertension [10]. Additionally, studies utilizing ambulatory blood pressure monitoring have reported that 37–75% of subjects with WCH progress to sustained hypertension over time [11, 12]. In children, the risks associated with WCH and the potential timeline for complications are not well understood, as cardiovascular morbidity is exceedingly rare. However, some studies suggest that the left ventricular mass index may be intermediate between normotensive and hypertensive children when accounting for BMI [7, 13]. Furthermore, similar to adults, obesity is often observed in children with WCH, which may increase the risk of progression to sustained hypertension [14]. Guidelines on pediatric hypertension recommend that at least three separate visits be conducted to confirm the diagnosis of hypertension in children [15]. However, conducting three separate visits is challenging in large-scale population screenings, and one-time screening was more commonly used. Therefore, considering the clinical significance and practical feasibility, a one-time blood pressure screening to identify occasional hypertension remains important.

Childhood obesity is a global problem, with an age-standardized prevalence of 5.6% in girls and 7.8% in boys in 2016 [16]. It has been known for some time that obesity is associated with HTN in both adults and children [17]. In addition to childhood obesity, size at birth and subsequent weight gain during early postnatal life are essential indicators of neonatal and adult health. It is well documented that small for gestational age (SGA, < 10th percentile birth weight for gestation) infants are facing increased risks of adverse developmental and adult health outcomes, including lower psychologic and intellectual performance, precocious puberty, type 2 diabetes, metabolic syndrome, obesity, and cardiovascular disease [18–20]. Previous studies have concluded that excessive weight gain and overweight in the first several years of life would also increase the risks of subsequent obesity and unfavorable metabolic outcomes in childhood, adolescence, and adulthood [21–23]. However, to our knowledge, no studies compared the effects or evaluated the joint effect of body weight at birth, infancy, and childhood on childhood HTN. There were also no studies focusing on the impact of weight status change from birth to infancy and currency on childhood BP.

Globally, 16% of infants are SGA at birth, ranging from 7% in industrialized countries to 41.5% in South Asia in 2010 [24]. Most SGA infants catch up with their appropriate gestational age (AGA, 10th ~ < 90th percentile birth weight for gestation) counterparts by exhibiting a faster weight and length gain rate, crossing upwards the percentiles on the growth charts. However, the health impact on catch-up growth following SGA was controversial, and the evidence was limited [25]. It has been suggested that catch-up growth following SGA is beneficial in a short time, but catch-up growth that occurs later than 2 years increases the risk of later obesity and non-communicable diseases [25, 26]; however, this evidence does not focus on individuals with SGA and has not been subject to systematic review and evidence appraisal.

Using the data of a population-based retrospective cohort study in China, the present study aimed to assess the prevalence of childhood occasional hypertension (OHTN) at 5 ~ 6 years old and its association with body size at birth and BMI at infancy and currency in full-term born children in China. We also compared the effects of catch-up growth before and after 2 years following SGA on childhood OHTN to seek a better growth pattern during early childhood for SGA infants.

## Methods

### Study sample

Tianjin is a metropolitan city in Northern China, ranking fourth in population size (15.6 million in 2017) among Chinese cities. It comprises 16 county-level administrative areas (including six central urban districts, one new urban district, four suburban districts, and five rural districts). Children’s health care in Tianjin is routine, with a three-tier care system consisting of (1) About 300 community-based health centers and 1700 kindergartens; (2) 16 district-level women and children’s health centers (WCHC) and other secondary obstetric hospitals; and (3) A city-level WCHC and other tertiary obstetric hospitals. In Tianjin, all children are given health examinations by the community-based health centers at birth, postnatal (< 42 days after birth), infancy, and early childhood, and then by the district-level or city-level WCHC after entering kindergartens. Information of health examinations for children begins with children’s birth, including information from newborns (date of birth, sex, gestational week of birth, birth weight, birth recumbent length, and Apgar score), postnatal period (names of the child and their parents, family history of diseases, feeding modalities, weight, and recumbent length), infancy (date of examination, weight, recumbent length before 24 months and height from 24 months, head circumference, number of teeth, and blood hemoglobin), and preschool (date of examination, weight, height, number of teeth, blood hemoglobin, and BP). Healthcare records have been collected and available electronically since 2009 [20]. We conducted a retrospective cohort study in Tianjin, China, based on the electronic data from the health care records.

**Fig. 1:**
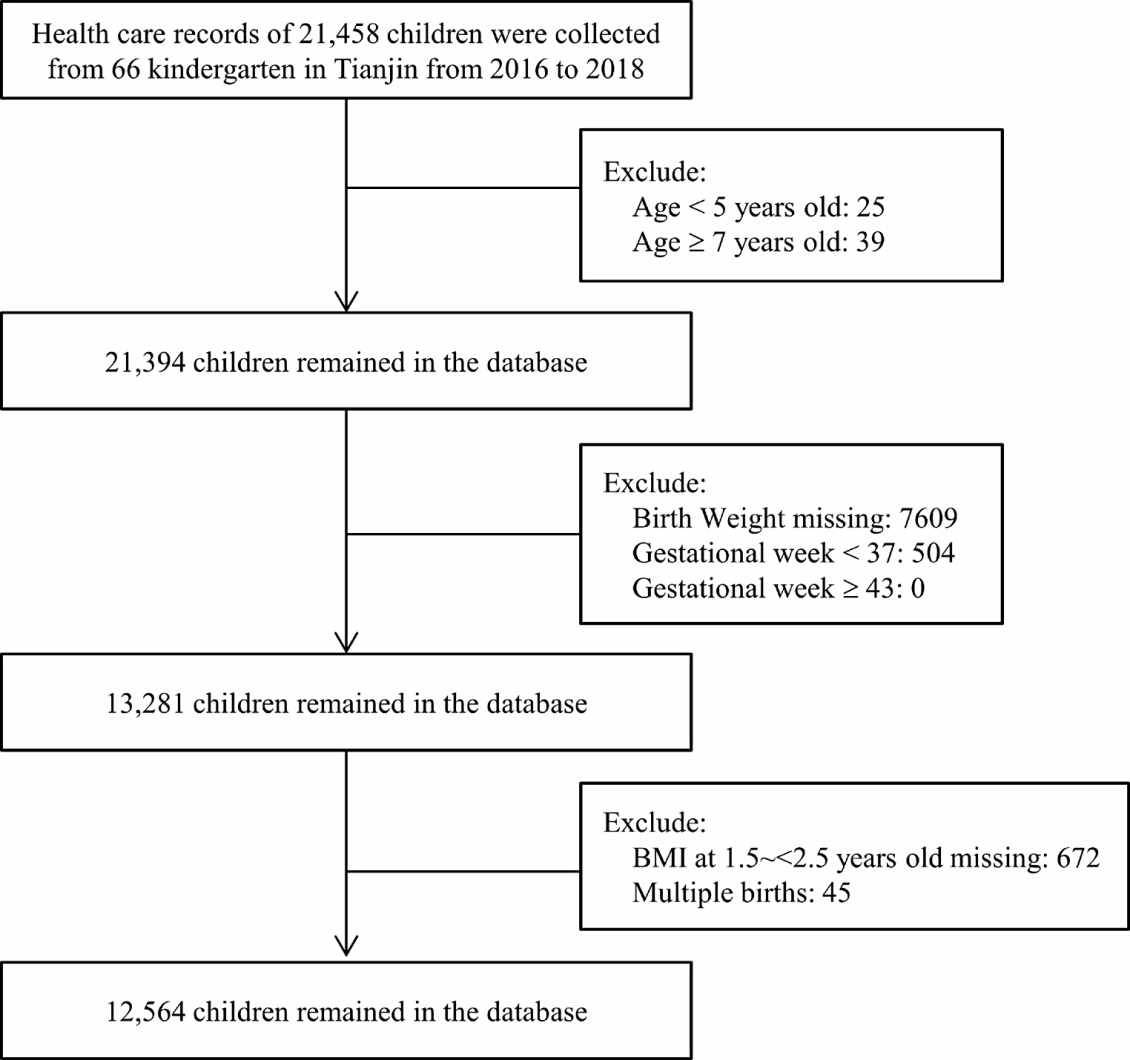
Detailed flow chart for the data cleaning procedure

We employed stratified cluster sampling to obtain a random sample of children in Tianjin, and 1 ~ 7 kindergarten schools were selected from each of the 16 districts. Between 2016 and 2018, 66 kindergartens were monitored, and 21,458 children at 5 ~ 6 years old finished the BP screening. Birth weight and BMI information at 2 years old (1.5 ~ < 2.5) and 5 ~ 6 years old (5.0 ~ < 7.0) were retrospectively collected from the electronic health care records of annual regular health examinations. The present analyses included 12,564 (58.6%) children after excluding 8,894 children with incomplete data (birth weight, gestational age, BMI at 2 years old), older than 7 years old, or younger than 5 years old, more than 43 or less than 37 weeks of gestational age, and multiple births (Fig. 1). There were no significant differences in gender composition (male: 52.8% vs. 52.2%, *P* = 0.369) but significant differences in age (6.11 ± 0.34 vs. 6.09 ± 0.31 years old) between children excluded and included in the present analysis. The Ethics Committee for Clinical Research of Tianjin Women and Children’s Health Center approved the study and analysis plan. Since this is a retrospective analysis of data routinely collected from children, written informed consent was not applicable. The Ethics Committee for Clinical Research of Tianjin Women and Children’s Health Center agreed to waive the need for written informed consent from all participants in our study.

### Measurements

The dataset for the present analysis was from electronic healthcare records of the annual regular health examination. The dataset included family information (paternal BMI, maternal BMI, family income, residential region), information at birth (delivery mode, birth weight, birth length and gestational age), infant feeding pattern within the first 6 months, information at 2 years old (1.5 ~ < 2.5) (age, weight and height/length) and 5 ~ 6 years old [age, weight, height, systolic blood pressure (SBP) and diastolic blood pressure (DBP)].

BP was measured using electronic sphygmomanometers (Omron HBP1300), certified by the Association for the Advancement of Medical Instrumentation (AAMI). Children were asked to empty their bladder and had at least 5 min rest before BP measurements. Then, they were seated with their legs uncrossed and right arm at the heart level. It was recommended that they be relaxed and keep quiet. An appropriate cuff was used according to the circumference size of the child’s right upper arm. The measurements were taken twice for each child at an interval of 1 min. If the difference between the two measurements ≥10 mm Hg, a third measurement was taken. The mean values of the last two readings were used for diagnosis and statistical analysis. The definition of hypertension in children and adolescents is based on the BP percentile, as the average SBP and/or DBP is ≥ 95th percentile (*P*_95_) for gender, age, and height according to the 2017 Chinese standards [27]. As HTN in children and adolescents should be determined from at least three separate visits [15] and BP was measured on only one occasion in this study, we defined those who met the diagnostic criteria as OHTN.

Children’s weight and length/height were measured at birth, 2 years old (1.5 ~ < 2.5), and 5 ~ 6 years old (5.0 ~ < 7.0). The age of the child (calculated as the difference between the date of measurement and birth) was recorded and included in the analysis. Using the standardized protocol, specially-trained pediatricians measured all children’s weight and length/height in light indoor clothing and without shoes. Weight was measured to the nearest 0.01 kg using a digital scale (TCS-60, Tianjin Weighing Apparatus Co., China). The length was measured to the nearest 0.1 cm using a recumbent length stadiometer (YSC-2, Beijing Guowangxingda, China). Standing height was measured to the nearest 0.1 cm using a stadiometer (SZG-180, Shanghai Zhengdahengqi, China). The electronic healthcare records were verified with repeated measurements previously [28]. The correlations between electronic healthcare records and repeated measurement data for children are 0.999 (for body weight) and 0.999 (for height/recumbent length), respectively.

Z scores (standard deviation [SD] scores) were calculated independent of sex and age– that is, (measurement minus population mean) /population SD. Birth weight z scores were calculated based on the 2014 Chinese child growth standards [29]. BMI z scores at 2 and 5 ~ 6 years old were calculated based on the World Health Organization (WHO) child growth standards (2 ~ 5 years old) [30] and WHO child growth reference (5 ~ 19 years old) [31].

### Groups

1) At birth, we focused on intrauterine growth. Newborns were divided into three subgroups according to birth weight Z scores: SGA (<-2SD), AGA (-2SD to 2SD), and large for gestational age (LGA) (≥2SD) [29].

2) At 2 years old, we focused on catch-up growth in the first 2 years after birth. Children were divided into three categories according to BMI Z scores at 2 years old: <10th centile (no catch-up growth), 10th to 75th centile (moderate catch-up growth), and ≥75th centile (excessive catch-up growth) in each birth weight subgroup [32]. The 75th percentile of Z-distribution was converted to 0.67 SD.

3) At 5 ~ 6 years old, we focused on childhood obesity. Children at 5 ~ 6 years old were divided into three categories: <-2SD (underweight), -2SD to 2SD (normal weight), and ≥2SD (obesity) [33] in each birth weight subgroup.

### Statistical analyses

The rates of OHTN were compared between different groups using the *chi-square* test. Logistic regression models were used to estimate the associations of birth weight groups and childhood BMI with OHTN at 5 ~ 6 years old in full-term born children. We included three models in the logistic analyses: Model 1 univariate analyses; Model 2 adjusted for paternal BMI, maternal BMI, family income, residential region, delivery mode, infant feeding pattern, sex, and age; Model 3 adjusted for variables in Model 2 and further adjusted for BMI at 2 and 5 ~ 6 years old (in birth weight groups analyses), or birth weight groups and BMI at 5 ~ 6 years old (in BMI at 2 years old analyses), or birth weight groups and BMI at 2 years old (in BMI at 5 ~ 6 years old analyses).

We stratified participants by birth weight groups (SGA, AGA, and LGA) to see the association of BMI at 2 and 5 ~ 6 years old with OHTN. The joint association of OHTN with birth weight groups, BMI at 2 and 5 ~ 6 years old were also tested, with the children who were AGA at birth, with BMI 10th ~ < 75th at 2 years old and BMI − 2SD ~ 2SD at 5 ~ 6 years old as the reference group. The criterion of statistical significance was < 0.05 (for two-sided tests). All statistical analyses used SAS for Windows, version 9.3 (SAS Institute, Cary, NC) and R 4.0 (R Foundation for Statistical Computing, Vienna, Austria) software programs.

## Results

### Incidence of occasional hypertension

Of the 12,564 children, 6,561 (52.2%) were boys and 6,003 (47.8%) girls. Overall, the rates of SBP ≥ P_95_, DBP ≥ P_95_, and OHTN at 5 to 6 years old were 10.6% (1,333/12,564), 10.0% (1,255/12,564) and 17.0% (2,133/12,564), respectively. Table 1 shows that children whose parents were overweight/obese, lived outside rural areas, who were boys, and delivered by cesarean section had higher incidences of OHTN (all *P* values < 0.05). There was no difference between different family income, infant feeding patterns, or birth weight groups (SGA, AGA, and LGA). The rates of hypertension were different in BMI categories. The OHTN rate at 2 years old was 16.4%, 15.5%, and 18.8% for BMI < 10th, 10th ~ < 75th, and ≥75th categories; at 5 ~ 6 years old, it was 8.9%, 15.1%, and 34.4% for BMI <-2SD, -2SD ~ < 2SD, and ≥2SD categories, respectively.

**Table 1:** Rates of occasional hypertension at 5 ~ 6 years old according to different categories of characteristics

	Sample Size	SBP ≥ *P*_95_		DBP ≥ *P*_95_		OHTN	
		Rate	*P* value	Rate	*P* value	Rate	*P* value
Paternal BMI			0.015		0.001		0.001
< 24 kg/m^2^	4,364	9.7		8.7		15.5	
≥24 kg/m^2^	8,200	11.1		10.7		17.8	
Maternal BMI			< 0.001		< 0.001		< 0.001
< 24 kg/m^2^	10,044	9.9		9.3		16.0	
≥ 24 kg/m^2^	2,520	13.3		12.6		20.8	
Family income			0.226		0.341		0.252
< 5,000 RMB	1,734	11.7		11.0		18.3	
5000 ~ < 10,000 RMB	5,402	10.6		9.8		16.8	
≥ 10,000 RMB	5,311	10.2		9.8		16.7	
Residential region			< 0.001		< 0.001		< 0.001
Urban	4,550	10.4		10.5		17.3	
Sub-urban	2,746	14.5		13.1		23.2	
Rural	2,625	5.5		8.2		11.1	
New urban	2,643	12.1		7.6		15.9	
Delivery mode			0.004		< 0.001		< 0.001
Vaginal delivery	4,912	9.6		8.5		15.0	
Cesarean section	7,652	11.2		11.0		18.2	
Infant feeding pattern			0.842		0.019		0.288
Exclusive breastfeeding	5,362	10.8		9.1		16.4	
Mixed feeding	6,684	10.4		10.7		17.4	
Exclusive formula feeding	434	10.8		10.4		18.2	
Sex			0.009		0.002		0.001
Boys	6,561	11.3		10.8		18.1	
Girls	6,003	9.9		9.1		15.7	
Age			0.172		0.006		0.023
5 ~ < 6 years	5,182	11.1		10.9		17.9	
6 ~ < 7 years	7,382	10.3		9.4		16.3	
Birth weight group			0.308		0.098		0.112
SGA	614	12.4		11.9		19.9	
AGA	9,765	10.5		10.1		16.9	
LGA	2,185	10.8		9.1		16.3	
BMI at 2 years old			< 0.001		0.013		< 0.001
< 10th	420	10.5		8.3		16.4	
10th ~ < 75th	6,539	9.2		9.4		15.5	
≥ 75th	5,605	12.3		10.8		18.8	
BMI at 5 ~ 6 years old			< 0.001		< 0.001		< 0.001
< -2SD	246	4.9		5.3		8.9	
-2SD ~ < 2SD	11,042	9.2		8.7		15.1	
≥ 2SD	1,276	24.1		21.9		34.4	

Occasional hypertension was defined as SBP ≥ *P*_95_ or DBP ≥ *P*_95_

SBP, systolic blood pressure; DBP, diastolic blood pressure; OHTN: occasional hypertension; BMI, body mass index; SGA, small for gestational age; AGA, appropriate for gestational age; LGA, large for gestational age; SD, standard deviation

According to the results in Table 1, we further analyzed the effects of birth weight and childhood BMI on OHTN (Table 2). Univariate analyses (Model 1) showed that children with a higher BMI (≥ 75th at 2 years old; ≥ 2SD at 5 ~ 6 years old) had a higher risk of OHTN [OR and 95% CI: 1.26 (1.15–1.39); 2.94 (2.59–3.42)]. After adjusting parents’ BMI, family income, residential region, delivery mode, infant feeding pattern, sex, and age (Model 2), high childhood BMI still contributed to the increased incidence of OHTN [OR and 95% CI: 1.20 (1.09–1.33); 2.93 (2.56–3.35)].

**Table 2:** Odds ratios and 95% confidence intervals of birth weight, BMI at 2 and 5 ~ 6 years old with occasional hypertension

	Model 1			Model 2			Model 3		
	SBP ≥ *P*_95_	DBP ≥ *P*_95_	OHTN	SBP ≥ *P*_95_	DBP ≥ *P*_95_	Occasional hypertension	SBP ≥ *P*_95_	DBP ≥ *P*_95_	OHTN
Birth weight groups									
SGA	1.21 (0.94–1.55)	1.20 (0.93–1.55)	1.22 (0.99–1.49)	**1.30 (1.01–1.68)**	**1.29 (1.00-1.67)**	**1.30 (1.06–1.61)**	**1.37 (1.06–1.78)**	**1.33 (1.03–1.73)**	**1.36 (1.10–1.68)**
AGA	1.00	1.00	1.00	1.00	1.00	1.00	1.00	1.00	1.00
LGA	1.04 (0.89–1.20)	0.89 (0.76–1.04)	0.95 (0.84–1.08)	0.95 (0.82–1.11)	**0.81 (0.69–0.96)**	**0.87 (0.76–0.99)**	0.87 (0.74–1.02)	**0.76 (0.64–0.89)**	**0.81 (0.71–0.92)**
BMI at 2 years old									
< 10th	1.16 (0.84–1.60)	0.88 (0.62–1.26)	1.07 (0.82–1.40)	1.10 (0.79–1.53)	0.86 (0.60–1.24)	1.04 (0.79–1.36)	1.22 (0.87–1.71)	0.93 (0.64–1.34)	1.13 (0.86–1.49)
10th ~ < 75th	1.00	1.00	1.00	1.00	1.00	1.00	1.00	1.00	1.00
≥ 75th	**1.38 (1.23–1.55)**	**1.18 (1.05–1.33)**	**1.26 (1.15–1.39)**	**1.34 (1.19–1.51)**	1.11 (0.98–1.25)	**1.20 (1.09–1.33)**	**1.18 (1.04–1.33)**	0.99 (0.87–1.12)	1.08 (0.98–1.19)
BMI at 5 ~ 6 years old									
< -2SD	**0.51 (0.28–0.91)**	0.58 (0.33–1.03)	**0.55 (0.35–0.86)**	**0.54 (0.30–0.97)**	0.64 (0.36–1.12)	**0.59 (0.38–0.91)**	**0.52 (0.29–0.95)**	0.61 (0.34–1.08)	**0.56 (0.35–0.87)**
-2SD ~ < 2SD	1.00	1.00	1.00	1.00	1.00	1.00	1.00	1.00	1.00
≥ 2SD	**3.15 (2.73–3.64)**	**2.95 (2.54–3.42)**	**2.94 (2.59–3.34)**	**3.23 (2.77–3.76)**	**2.82 (2.42–3.30)**	**2.93 (2.56–3.35)**	**3.15 (2.70–3.69)**	**2.89 (2.46–3.39)**	**2.93 (2.56–3.36)**

Occasional hypertension was defined as SBP ≥ P_95_ or DBP ≥ P_95_

Model 1 univariate analyses;

Model 2 adjusted for paternal BMI, maternal BMI, family income, residential region, delivery mode, infant feeding pattern, sex, and age;

Model 3 adjusted for variables in Model 2 and further adjusted for BMI at 2 and 5 ~ 6 years old (in birth weight groups analyses), or birth weight groups and BMI at 5 ~ 6 years old (in BMI at 2 years old analyses), or birth weight groups and BMI at 2 years old (in BMI at 5 ~ 6 years old analyses)

SBP, systolic blood pressure; DBP, diastolic blood pressure; OHTN: occasional hypertension; SGA, small for gestational age; AGA, appropriate for gestational age; LGA, large for gestational age; BMI, body mass index; SD, standard deviation

When birth weight groups, BMI at 2 and 5 ~ 6 years old entered the model simultaneously (Model 3), the multivariate-adjusted ORs (95% CIs) of OHTN at 5 ~ 6 years old were 1.36 (1.10–1.68), 1.00 and 0.81 (0.71–0.92) based on different groups of birth weight (SGA, AGA, and LGA); 1.13 (0.86–1.49), 1.00 and 1.08 (0.98–1.19) based on different categories of BMI at 2 years old (< 10th, 10th ~ < 75th and ≥75th); and 0.56 (0.35–0.87), 1.00 and 2.93 (2.56–3.36) based on different categories of BMI at 5 ~ 6 years old (<-2SD, -2SD ~ < 2SD and ≥2SD), respectively.

### Occasional hypertension under different weight statuses

**Fig. 2:**
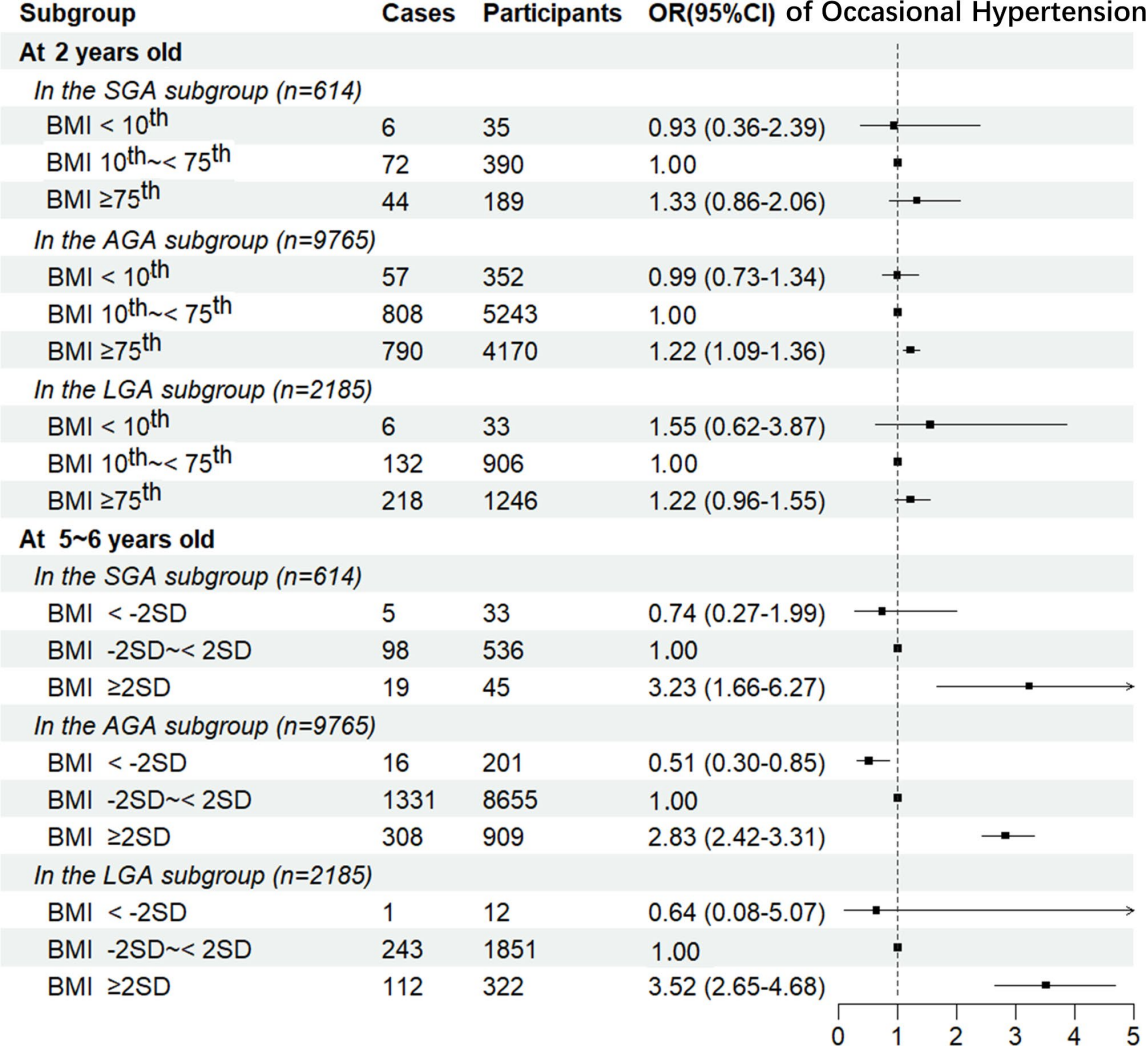
Odds ratios and 95% confidence intervals of BMI to age-specific occasional hypertension. Adjusted for paternal BMI, maternal BMI, family income, residential region, delivery mode, infant feeding pattern, sex, and age

In the subgroup analysis of birth weight, the association of BMI at 2 and 5 ~ 6 years old with OHTN at 5 ~ 6 years old was shown in Fig. 2. BMI at 2 years old (< 10th, 10th ~ < 75th and ≥75th) was significantly associated with OHTN in children who were AGA at birth [ORs and 95% CIs: 0.99 (0.73–1.34), 1.00 and 1.22 (1.09–1.36). However, the association was not significant in children who were SGA or LGA at birth. Being obese at 5 ~ 6 years old (BMI ≥ 2SD) would significantly increase the risk of OHTN at 5 ~ 6 years old regardless of birth weight [OR and 95% CI: SGA 3.23 (1.66–6.27); AGA 2.83 (2.42–3.31); LGA 3.52 (2.65–4.68)].

### Joint association of OHTN with birth weight and childhood BMI

**Fig. 3:**
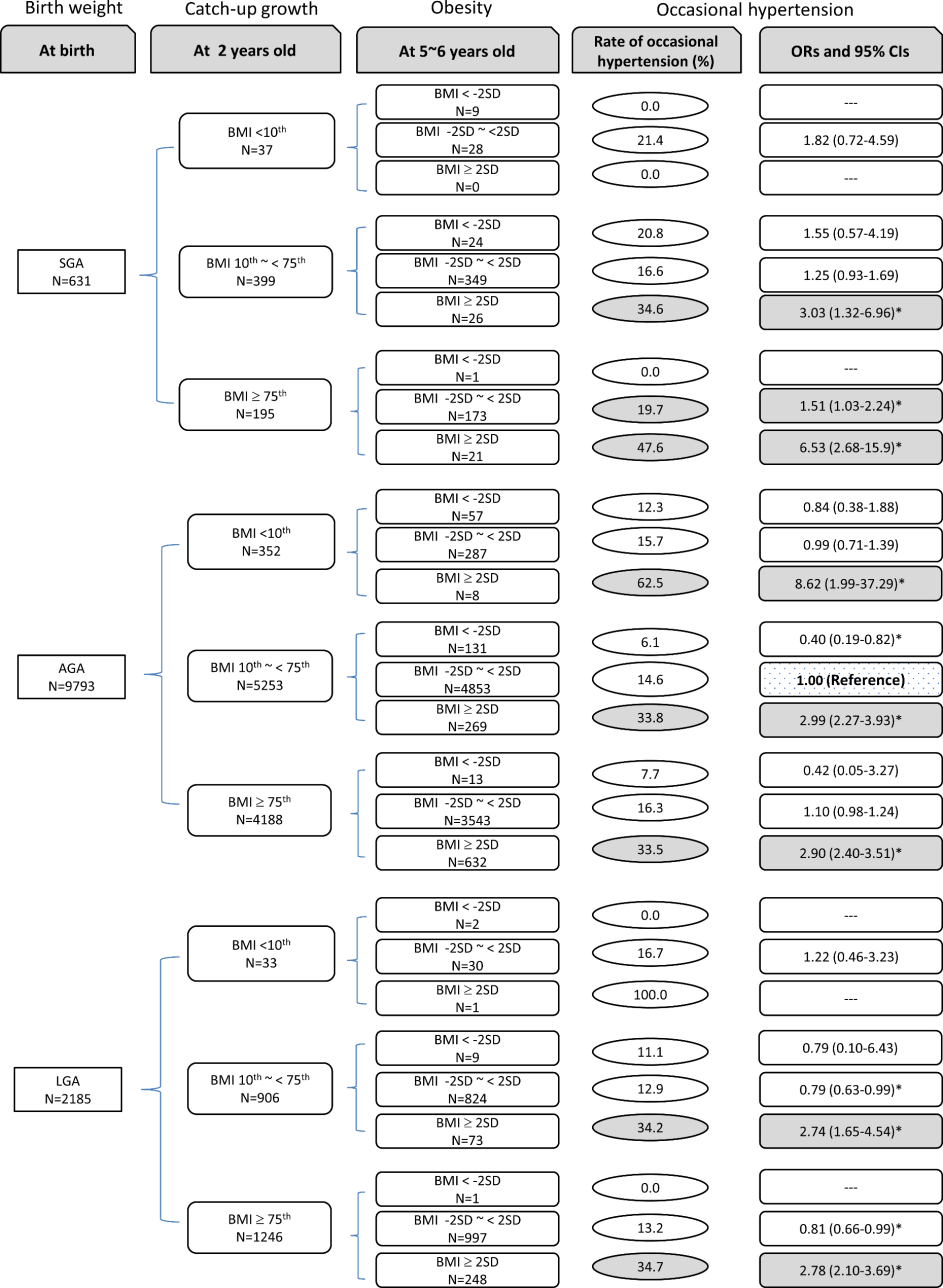
Odds ratios of occasional hypertension by the joint effect of birth weight groups and BMI. Adjusted for paternal BMI, maternal BMI, family income, residential region, delivery mode, infant feeding pattern, sex, and age. * there were significant differences compared with the children who were AGA at birth, with BMI 10th ~ < 75th at 2 years old and BMI − 2SD ~ < 2SD at 5 ~ 6 years old

The joint association of OHTN at 5 ~ 6 years old with birth weight groups, BMI at 2 and 5 ~ 6 years old were listed in Fig. 3. The children who were AGA at birth with BMI 10th ~ < 75th at 2 years old and BMI − 2SD ~ 2SD at 5 ~ 6 years old were defined as the control group.

In the SGA group, children with BMI 10th ~ < 75th at 2 years old and BMI ≥ 2SD at 5 ~ 6 years old had increased risk of OHTN at 5 ~ 6 years old [OR and 95% CI: 3.03 (1.32–6.96)]; children with BMI ≥75th at 2 years old and BMI − 2SD ~ 2SD at 5 ~ 6 years old had increased risk of OHTN at 5 ~ 6 years old [OR and 95% CI: 1.51 (1.03–2.24)]; and co-presence of over catch growth at 2 years old (≥75th) and obesity at 5 ~ 6 years old increased the OR to 6.53 (2.68–15.90).

In the AGA group, the risk of OHTN at 5 ~ 6 years old was significantly increased in each obesity group (BMI ≥ 2SD at 5 ~ 6 years): BMI < 10th at 2 years old [OR and 95% CI: 8.62 (1.99–37.29)]; BMI 10th ~ < 75th at 2 years old [OR and 95% CI: 2.99 (2.27–3.93)]; BMI ≥75th at 2 years old [OR and 95% CI: 2.90 (2.40–3.51)].

In the LGA group, children with catch-up growth and co-presence of obesity at 5 ~ 6 years old had increased risk of OHTN: BMI 10th ~ < 75th at 2 years old and BMI ≥ 2SD at 5 ~ 6 years old [OR and 95% CI: 2.74 (1.65–4.54)]; BMI ≥75th at 2 years old and BMI ≥ 2SD at 5 ~ 6 years old [OR and 95% CI: 2.78 (2.10–3.69)].

## Discussion

Obesity was a vital risk factor for childhood OHTN regardless of birth weight or catch-up growth. Childhood OHTN had the strongest association with current BMI, followed by size at birth and then BMI at 2 years of age. For SGA infants, both excessive catch-up growth and obesity were associated with a higher risk of OHTN. In other words, moderate catch-up growth before 2 years old and a subsequent normal weight at 5 ~ 6 years old may prevent childhood OHTN in SGA.

Based on a population-based retrospective cohort study from China, the present study showed that the prevalence of OHTN in preschool children at 5 ~ 6 years old was 17.0%, and boys had a higher risk of hyperlipidemia than girls. Many epidemiological surveys have focused on pediatric BP, but most were school-based and conducted among school-aged children and adolescents, and data on preschool children was limited. Besides, most of the previous school-based surveys had BP measurement on only one occasion, and the results varied greatly from 3.1 to 16.7% [9–14]. As the level of BP is dynamic and can be easily affected by many factors, guidelines on pediatric HTN have recommended that at least three separate visits should be taken to confirm the diagnosis of HTN in children [15]. A meta-analysis based on 47 articles showed that the global prevalence of HTN in children and adolescents aged < 19 years was 4.00% based on BP measurements in at least three separate visits [34]. The present study had BP measured on only one occasion, so we cannot know the actual HTN prevalence from our study if defined after three separate visits. However, according to the previous research, high BP tends to fall on subsequent measurement, and only less than 1/4 of the children defined as OHTN would be the target children with HTN: it dropped out substantially from visit 2 to visit 3, and around 56.7% and 75.9% of these hypertensive ones at visit 1 returned to normal BP at visit 2 and visit 3, respectively [35]. Thus, we conjecture that the actual HTN prevalence of our study, if defined after three separate visits, might be similar to 4%. HTN, which once was considered a rare disease in children, is now actually a significant public health problem and has migrated to preschool age. Our result highlighted the importance of performing BP screening and early intervention in preschool children to reduce the adverse effects of HTN.

It has been documented that size at birth and childhood weight are associated with BP levels in childhood. Size at birth is an essential indicator of neonatal and childhood health. Previous studies suggested that subjects with low birth weight groups might be associated with the defect in insulin sensitivity [36] and deficient pancreatic β-cell function [37], which may result from fetal adaptation to an adverse intrauterine environment during a critical period, leading to long-lasting programming of fetal gene expression [38]. It was found that 63% of children at the age of 2 years with very low birth weight had raised blood pressure, and compared with term AGA children, VLBW and term SGA children had a higher prevalence of metabolic syndrome components in early childhood [39]. In our study, the risk of having OHTN was higher in children with SGA, consistent with previous studies. Several studies have confirmed a clear association between elevated blood pressure and excessive adiposity storage. Meta-analysis showed that the prevalence of childhood hypertension was higher in the obesity and overweight group compared with children with normal weight, and subjects with obesity have a 3.5-fold increased risk of having hypertension [7, 21]. Excessive visceral fat storage is associated with hormonal, inflammatory, and endothelial alterations, stimulating the sympathetic nervous system, endothelial dysfunction, and increased sodium retention. These mechanisms may lead to elevated blood pressure levels [7]. The present study confirmed that BMI groups were associated with blood pressure levels, and children with obesity were at increased risk of having OHTN.

DOHaD (Developmental Origins of Health and Disease) highlights the fetal origins of metabolic syndrome and the vital impact of postnatal growth and other factors on later metabolic outcomes. If SGA infants remain underweight without catch-up growth, the adverse programming effects of fetal growth restriction will persist. Most SGA infants tend to show “catch-up growth” after growth, and approximately 85% of SGA infants achieve appropriate catch-up growth in height [40]. It has been suggested that faster growth over the first 2 years could offset the adverse programming effects of fetal growth restriction and low birth weight groups (LBW). For example, in the Helsinki and New Delhi Birth Cohorts, individuals with LBW who had lower BMI in the first 2 years were noted to be at the highest risk of T2DM and CVD [41, 42]. However, such catch-up growth has recently been linked to an increased risk of later adiposity, insulin resistance, and cardiovascular disease, as SGA infants tend to gain weight more rapidly than height during the early postnatal period [43, 44]. In 2017, a systematic review involving eight studies in seven cohorts demonstrated that catch-up growth following LBW may benefit individuals in the short term and have adverse population health impacts in the long term. However, the evidence is limited, considering the low quantity and quality of evidence [45]. Another review by *Victora* pointed out that catch-up growth following SGA before the age of 2 years is beneficial for long-term health outcomes. However, catch-up growth that occurs later than 2 years increases the risk of later obesity and non-communicable diseases, but this evidence has not focused on individuals with LBW and has not been subject to systematic review and evidence appraisal [26]. Instead of weight or height catch-up growth, the present study focused on BMI catch-up growth at around 2 and 5 ~ 6 years old. We found that BMI at 2 years was not associated with children born SGA, while BMI at 5 ~ 6 years was significantly associated with childhood dyslipidemia. Results were consistent with *Victora’s* review, and we suggested that SGA infants should achieve catch-up growth before the age of 2 years, considering the negative effect on BP metabolism and potential benefit on neural intelligence development; however, after 2 years old, SGA children should achieve normal weight without being underweight or obesity to reduce the risk of childhood OHTN.

The major strength of this study was that we reported the prevalence of OHTN in children aged 5 ~ 6 years in China based on population-based data. Furthermore, we focused on the joint effect of birth weight, catch-up growth, and preschool-age obesity on childhood hypertension. At the same time, we have some limitations. First, 41.3% of the children screened were excluded as they lacked some of the critical information. Though we compared the general characteristics between the included and excluded children, we could not exclude the possibility that the observed effect sizes in our study had departed from the actual value. Second, our study examined only TC and TG as indicators of altered BP metabolism. This results in differences in the prevalence of OHTN between our research and other studies. In the future, we might also test blood low-density lipoprotein and high-density lipoprotein concentrations to evaluate children’s BP metabolism comprehensively. Third, although our analyses have included some potential prediction factors, other unmeasured factors were associated with the risks of obesity and OHTN, which could confound our results.

## Conclusion

SGA infants should achieve moderate catch-up growth before the age of 2 years, considering the negative effect on BP metabolism and the potential benefits of neural intelligence development. Both AGA and LGA should also avoid childhood obesity to reduce the risk of hypertension.

## Data Availability

The datasets generated and/or analyzed during the current study are not publicly available because the data in this study are public health data and are protected by government security laws and regulations but are available from the corresponding author upon reasonable request.

## Abbreviations

HTN: Hypertension
BP: Blood pressure
SGA: Small for gestational age
AGA: Appropriate for gestational age
OHTN: Occasional hypertension
BMI: Body mass index
WCHC: Women and children’s health centers
SBP: Systolic blood pressure
DBP: Diastolic blood pressure
SD: Standard deviation
WHO: World Health Organization
LGA: Large for gestational age
AAMI: Association for the Advancement of Medical Instrumentation
OR: Odds ratio
CI: Confidence intervals
